# Feasibility and Acceptability of Formats in a Comparative Effectiveness Trial of a Preventive Parenting Program

**DOI:** 10.1007/s10826-025-03016-z

**Published:** 2025-02-05

**Authors:** Gretchen Buchanan, Tori Simenec, Qiyue Cai, Abigail Gewirtz

**Affiliations:** 1 Redleaf Center for Family Healing, Department of Psychiatry, Hennepin Healthcare Research Institute, Minneapolis, MN USA; 2 Department of Family Medicine and Community Health, University of Minnesota Medical School, Minneapolis, MN USA; 3 Institute of Child Development, University of Minnesota Twin Cities, Minneapolis, MN USA; 4 Department of Psychology, Arizona State University, Tempe, AZ USA; 5 Paul Baerwald School of Social Work & Social Welfare, Hebrew University of Jerusalem, Jerusalem, Israel

**Keywords:** Parenting programs, Military parenting, Telehealth, Comparative effectiveness, Program format acceptability

## Abstract

Parenting can be challenging, and military parents face additional family stressors related to relocations and deployments. ADAPT4U is an evidence-based preventive parenting program specifically designed for military parents of school-aged children. This study examines the feasibility and acceptability of in-person group and telehealth versions of the program. We used quantitative and qualitative data in a concurrent embedded design. Quantitative data were analyzed using SPSS with regressions and ANOVAs. Qualitative data were thematically coded by multiple authors and then a consensus process was undertaken. Both conditions were highly satisfactory for participants, with in-person group rated slightly higher. Families were more likely to attend telehealth than group, both at all and for at least 50% of sessions. Telehealth participants rated more highly: helpfulness, the facilitator was understanding and responsive, and they would participate in a future parenting program based on their experience. Qualitative results reflected positive experiences both with the content and facilitators, and concerns or recommendations that often were directly addressed by the other format (e.g., suggestions by in-person group participants for a telehealth format). Consistent themes across formats included wanting children to be involved in the program and more follow-up after the end of the program. The ADAPT4U program is highly acceptable to participants, and providing multiple format options (in-person group and individual telehealth) will likely make it more feasible for parents to participate in a way that works best for them.

## Highlights

- Both formats of the intervention were highly satisfactory, with in-person rated higher.
- Attendance was more likely in the telehealth format.
- Offering parents multiple formats of the same program will likely improve reach, since different groups or types of parents might be looking for different features in a program.

## Background

Preventive parenting programs fall into several categories: universal, selective, and indicated. These types of programs aim to build parents’ skills in effective parenting, such as positive discipline strategies and monitoring children’s activities, and many are based on the Social Interaction Learning Theory and its operationalization as Parent Management Training – Oregon Model (PMTO, now known as GenerationPMTO; Forgatch & Patterson, 2010). Many of these interventions have shown effectiveness and feasibility in delivery, yet widespread dissemination continues to be limited due to reasons such as stigma, no clear access points, parents’ trust in sponsoring organizations, and funding issues (Leslie et al., 2016), as well as barriers to participation such as competing priorities and logistical issues (Rostad et al., 2018).

## The Need for Parenting Programs among Military Families

Over 20 years of post 9/11 conflicts, millions of US service members were deployed to Iraq and Afghanistan, and almost one third of those (30.9%) were married with children, with an additional 5.8% single parents (Department of Defense, 2023). More than 2 million children experienced the deployment of a parent to the conflicts, and more than 1.5 million children currently have a parent serving (Department of Defense, 2023). Military families are resilient and committed, yet the stressors associated with deployment to war, as well as routine military transitions (e.g. Permanent Changes of Station/PCS that require many service members to move every three years) are significant. Military parents and their children can struggle with emotion dysregulation related to the uniquely stressful experiences of deployment (meaning one parent is absent for an extended period of time and the other parent must function as a solo parent), parental experiences of combat and increased risk of trauma response, or other military experiences (Gewirtz et al., 2017). Against this backdrop, supports for partner, family, and parenting success can strengthen adult and child adjustment in military families (Cai et al., 2023; DeGarmo et al., 2023; De Voe et al., 2017; Gewirtz, 2018; Gewirtz et al., 2016, 2017, 2018; Kearney et al., 2012; Kritikos et al., 2019; Walsh et al., 2014; Zhang et al., 2023).

## The Need to Compare Formats

Because some barriers to engaging in parenting programs could be overcome through online formats, such as transportation and even the potential for universal access to pre-recorded or static content, it seems reasonable to convert parenting programs generally to this format. However, there may be considerations for keeping programs in-person, such as the potential for greater effectiveness, social support, etc. Desires and needs of parents should be considered to facilitate engagement.

The COVID-19 pandemic further emphasized the pressing need for flexible and digital options for care to support parents as they navigate parenting through highly stressful circumstances. Some research points to technology-based parenting interventions as effective strategies to improve parenting behavior, parenting knowledge, and self-efficacy with an average participant completion rate of 78.3% (Corralejo & Domenech Rodríguez, 2018; Breitenstein et al., 2014). Telehealth-delivered interventions also appear to be noninferior to in-person services, and may help overcome some barriers parents often face to attending in person (Comer et al., 2017; Mogil et al., (2022); Traube et al., 2020).

One noninferiority trial (Engelbrektsson et al., 2023) and a recent meta-analysis (Leijten et al., 2024) indicate that both formats are equally effective. Engelbrektsson et al., (2023) found that parents generally preferred in-person, group programming over online, self-directed/individual programming, though both were rated highly. In a recent meta-analysis, Leijten et al., (2024) found similar satisfaction with formats, with a trend toward more satisfaction with online supports. Due to both the equal effectiveness and the lack of clarity on satisfaction/acceptability of formats, further exploration of acceptability and feasibility are needed. According to Proctor et al., (2011), acceptability (or satisfaction) is the perception among implementation stakeholders that an intervention is satisfactory or palatable, and feasibility is the extent to which an intervention can be used or or implemented in a given setting. These two implementation outcomes can have a significant impact on the actual use of an intervention; an intervention may be extremely effective but if it is not acceptable to participants and is not seen as feasible to implement, it will simply sit on a shelf. In fact, parent engagement and satisfaction have been shown to be more important than fidelity/implementation quality in terms of contributing to desired program effects (Giannotta et al., 2019). Because of the differences in attendance by format and because engagement is known to be an issue with parenting interventions (Chacko et al., 2016), feasibility and acceptability are important considerations.

## Intervention Description

ADAPT4U is the first empirically-validated parenting program designed for military families with school aged children and rigorously tested via randomized trials. ADAPT4U was originally developed as an in-person group-based parenting preventive intervention with home practice assignments and online tools to support learning and engagement between group sessions. This mindfulness-infused and trauma-informed parenting program delivered six core skills: teaching through encouragement, positive involvement, family problem-solving, monitoring and supervision, emotion socialization (emotion regulation and coaching skills), and effective limit setting). More details of the intervention are described in the Methods section.

The original program was tested in a randomized controlled trial with 336 military families with a parent deployed to Iraq or Afghanistan. Using intent-to-treat analyses, the 14-week group intervention was shown to improve effective observed parenting skills, parental efficacy, and parent and child adjustment from baseline (pre-intervention) to 12 months and 24 months post baseline with moderate effect sizes (Cohen’s d = 0.3–0.4;Gewirtz et al., 2018; (DeGarmo & Gewirtz, 2018; Piehler et al., 2018; Zhang et al., 2018). The program was extended, consistent with a public health approach, by adding different formats to vary intervention dosage and intensity while increasing the accessibility. This was accomplished through introducing two additional digital formats: telehealth and self-guided online program versions. Moreover, consistent with this approach, the formats were varied – the telehealth modality offered (1:1) virtual delivery while the in-person modality was delivered via multi-family groups. In Gewirtz et al., (2024), the telehealth format was found to be noninferior to the in-person group format, and both were more effective than the online self-directed format. In Gewirtz et al., (2024), attendance analyses indicated that participants attended telehealth and self-directed online formats significantly more than in-person group and provided detailed information regarding potential moderators. Slightly more than half of families randomized to in-person group attended at least once, whereas about three-quarters of those randomized to the other two formats engaged at least once. This study extends the outcomes and attendance analyses to explore reasons for these differences.

## The Current Study

The present study aims to compare the feasibility and acceptability of the telehealth format and original group format in the context of a randomized comparative effectiveness trial of three formats of the program. Given the conflicting state of the current literature, we do not have a priori hypotheses. We utilized a concurrent embedded mixed methods design to explore both implementation outcomes.

## Methods

### Design

[BLINDED] is a three-group, two-site randomized comparative effectiveness trial funded by the U.S. Department of Defense (Gewirtz et al., 2014). Families (*N* = 244) were randomly assigned to participate in one of three modalities of the [BLINDED] program: in-person group-based (*N* = 95); individual telehealth (*N* = 71); and individual self-directed online (*N* = 78). Both the group and telehealth conditions were also given access to the online program. Families participated in the study through four interview periods: pre-intervention baseline and post-intervention follow-ups at 6, 12, and 24 months. The present study describes data gathered during the intervention delivery period (baseline to posttest). The study was approved by the University of Minnesota IRB.

### Participants

Eligibility requirements for the study included having a child age 5–12 in the home, and at least one parent with an overseas deployment to Operation Iraqi Freedom or Operation Enduring Freedom. Participants were recruited in two Midwestern states through the National Guard, Veterans Affairs Medical Centers, and snowballing from previous participants. Recruitment activities included sending flyers with the study description, website URL, and phone number to partner organizations for distribution. The total sample included 244 families, with 191 fathers and 219 mothers participating; full demographics are listed in Table 1. Fathers averaged 37.6 years old (range = 25–54, *SD* = 5.8 years) and mothers averaged 35.9 years old (range = 24–56, *SD* = 5.7 years); target children averaged 7.7 years old (range = 4–12, *SD* = 2.3 years). Most parents identified as White (88.1% mothers, 86.4% fathers), then Black (4.6%, 7.3%), multiracial (3.2%, 1.6%), Asian/Asian-American (1.4%, 1.6%), Native American/Alaska Native (1.4%, 1%), Native Hawaiian/Pacific Islander (0.5%, 0%). Ninety-five percent of mothers and 92% of fathers identified as non-Hispanic. Over half (56%) of mothers and 40% of fathers had earned at least a bachelor’s degree. Annual family income mean was between $51,000 and $80,000.

**Table 1:** Sample demographics

		*M*	*SD*								
Age (years)											
- Dad^*^		37.6	5.8								
- Mom		35.9	5.7								
- Child (target)		7.7	2.3								
		Dad		Mom				Dad		Mom	
		*N*	%	*N*	%			*N*	%	*N*	%
Families	(*N* = 244)	191		219							
Marital status						Relationship to child					
	Married	165	86.8	181	83.0		Biological parent	169	88.5	204	93.2
	Divorced	11	5.8	16	7.3		Stepparent	16	8.4	8	3.7
	Separated	1	0.5	2	0.9		Adoptive parent	2	1.0	4	1.8
	Co-habiting/Domestic partnership	5	2.6	9	4.1		Co-habiting partner	4	2.1	3	1.4
	Single, not living with partner	8	4.2	10	4.6	Parent race					
Employment							Caucasian/White	165	86.4	193	88.1
	Employed, full time	149	78.0	117	53.4		African-American/Black	14	7.3	10	4.6
	Employed, part time	7	3.7	35	16.0		Native Hawaiian/PI	0	0	1	0.5
	Retired, no secondary occupation	9	4.7	1	0.5		Asian/Asian-American	3	1.6	1	1.4
	Retired with secondary occupation	1	0.5	2	0.9		Native American/Alaska Native	2	1.0	3	1.4
	Homemaker	4	2.1	43	19.6		Multiracial/Biracial	3	1.6	3	3.2
	Student and employed	1	0.5	10	4.6		Prefer not to answer/don’t know	4	2.1	7	0.9
	Student and not employed	5	2.6	7	3.2	Parent ethnicity					
	Unemployed	15	7.9	4	1.8		Hispanic/Latino	9	4.7	10	4.6
							Non-Hispanic/Non-Latino	176	92.1	207	94.5
							Prefer not to answer/don’t know	6	3.1	2	0.9
Parent education						Military service					
	Some high school	1	0.5				No	9	4.7	142	64.8
	GED	4	2.1	2	0.9		Yes	182	95.3	77	35.2
	High school diploma	10	5.2	8	3.7	Deployed overseas					
	Some college	59	30.9	51	23.3		Yes	180	94.2	60	27.4
	Associate degree	41	21.5	36	16.4		No	11	5.8	159	72.6
	Bachelor’s degree	50	26.2	83	37.9	Times deployed overseas					
	Master’s degree	20	10.5	37	16.9		1	51	28.7	39	63.9
	Doctoral/prof. degree	6	3.1	2	0.9		2	63	35.4	17	27.9
Family annual income							3	40	22.5	3	4.9
	Less than $25,000	3	1.6	8	3.7		4	10	5.6	1	1.6
	$26,000 to $50,000	40	20.9	40	18.3		5	7	3.9	0	-
	$51,000 to $80,000	56	29.3	69	31.6		6	3	1.7	1	1.6
	$81,000 to $100,000	33	17.2	36	16.5		7	3	1.7	0	-
	$101,000 to $120,000	34	17.8	36	16.4		10	1	0.6	0	-
	$121,000 to $150,000	18	9.4	21	9.6						
	$151,000 or more	7	3.6	9	4.1						

^*^Significantly different by condition

Regarding military service, 95.3% of fathers and 35.2% of mothers had served in the U.S. military. For fathers, 94.2% had been deployed overseas (range 0–8 times, *M* = 1.88, *SD* = 1.1) for an average 19.6 months total (*SD* = 11.9 months); for mothers, 27.4% had been deployed overseas (range = 1–6 times, *M* = 1.51, *SD* = 0.89) for an average 13.3 months total (*SD* = 8.5 months).

Families randomized to the three groups did not differ on control variables, except that families randomized to the online condition had fathers with significantly higher education levels compared to the group condition (*p* = 0.02). This variable was therefore included in all subsequent analyses to manage the difference.

### Procedures

Upon receiving recruitment materials, interested participants were directed to an anonymous screening questionnaire to determine eligibility, followed by collection of contact information and informed consent from each participating parent. Participants agreed to randomization to one of the three ADAPT4U modalities. Project staff contacted families to provide information about randomization, and for facilitator-delivered conditions (telehealth and group) a facilitator contacted the family with scheduling information. Further information about this process is documented in Gewirtz et al., 2024. For the purpose of this study, we will not describe the study itself but rather the procedures for acceptability measurement. After each group session, participants were asked to fill out a paper form, while after each telehealth session, participants were asked to log into their account and complete an online assessment.

### Conditions

Following participation in the baseline assessment, families were randomized into one of the three ADAPT4U formats: in-person group, individual telehealth, and individual self-directed online. In the group condition, families were grouped together based on location and time entering the trial. Families in the online modalities (telehealth and self-directed) were also assigned to cohorts to manage equivalence in analysis. Each session of the facilitator-delivered conditions was videorecorded for both fidelity monitoring and for facilitator coaching. Facilitators in both conditions received biweekly coaching. For the group format, skills were delivered by 2–3 facilitators in 14 weekly sessions lasting two hours each with six to 10 single or two-parent families. The telehealth format was identical to the group format (same material) but delivered in a (1:1) virtual (WebEx) format in hourly sessions by a single facilitator to a single family (1–2 parents). All families had access to the online program which provided materials to review and consolidate skills. Both facilitated formats (telehealth and group) involved active teaching, discussion and role play, and followed a standard format: greetings and review of the home practice from the prior week, introduction and practice of a new parenting skill, and preparing parents to try out the skill at home.

#### Group

The group facilitator called the family for orientation, addressing concerns or barriers to participation, describing confidentiality and its limits (harm to self or others or child maltreatment), and describing the program. Groups were 14 weeks long with meetings once a week, two hours each, and took place at community locations (e.g., schools, libraries) in the local area. Group facilitators were rigorously trained and some had previously delivered ADAPT4U or other PMTO parenting groups with high retention and acceptability. Between group sessions, participants were encouraged to engage with the ADAPT4U website, which had online content and a place for participants to upload home practice assignments. See Gewirtz et al., (2011) for detailed program content.

#### Telehealth

An [BLINDED] facilitator called the family for orientation, describing confidentiality and its limits (harm to self or others or child maltreatment), and describing the program. Participants were directed to the ADAPT4U website which was used to provide home practice assignments that participants then uploaded, as well as online viewing of videos demonstrating skills taught in sessions. Telehealth engagement was also 14 weeks long with meetings once a week, one hour each.

#### Self-directed online

Participants were directed to the ADAPT4U website, which provided ten online sessions including video demonstrations of the ADAPT4U skills, audio mindfulness exercises, and handouts with summaries and home practice information. Online-only condition participants received eleven weekly “check in” emails from an ADAPT4U facilitator to address any questions and check in on home practice completion. Parents were encouraged to respond to emails with progress, questions, and to troubleshoot.

### Measures

#### Demographics

Demographics gathered included child and parent (caregiver) age, child and parent sex, parent marital status, race/ethnicity, employment, education, military service and deployment experience, family annual income, and the caregiver relationship to child.

#### Feasibility

Feasibility was measured through engagement and retention measures. *Engagement* was measured by comparing the proportion of those who attended at least one session or completed one module to the number of invited participants. *Retention* was measured by comparing the proportion of invited participants to those who attended/completed 50% or more of the sessions/modules and those who completed the final session/module.

#### Acceptability of weekly sessions

Acceptability was measured for telehealth and group modalities (not for self-directed online) via a weekly 20-item questionnaire (19 items for telehealth), which was based on a participant feedback questionnaire originally developed for PMTO interventions (Forgatch & DeGarmo, 1999) and modified for ADAPT4U (Gewirtz et al., 2014). Items on the questionnaire use 5-point Likert scale ratings (for participation questions: 1 = *not at all* to 5 = *very much;* for home practice questions: 1 = *not applicable*, 2 = *not at all* to 6 = *very much*). First, answers to each question were described separately by mean and standard deviation. Then, a factor analysis and subsequent scale reliability tests were conducted for telehealth and group separately, for each session. Two subscales with reliability across sessions were included in the final analysis.

For the telehealth condition, three subscales were derived: *Participant acceptability* (ten items; agreed with the main ideas, facilitator encouraged my participation, felt open to new information, actively participated, pleasantly humorous things happened, paid careful attention, facilitator seemed to understand me, I like my facilitator, information was helpful, enjoyed today’s session; alphas ranged from 0.84 to 0.92); *Home practice acceptability* (5 items; home practice was helpful, did the homework assignment, was successful with the home practice, child(ren) responded well, assignment fits in well with my family; alphas ranged from 0.87 to 0.96), and *Participant barriers* (subscale did not have adequate reliability and therefore was dropped; items included: I felt angry or irritable, technology was a barrier in this session, I felt sad/down/depressed, the home practice was hard to do).

For the group condition, factor analysis initially produced four factors, but two were poor quality. Restricting this analysis to three factors, a similar pattern to the telehealth questionnaire emerged. The three subscales were: *Participant acceptability* (eleven items; agreed with the main ideas, leader(s) encouraged group participation, felt open to new information, actively participated, pleasantly humorous things happened, paid careful attention, felt accepted by other group members, group leader(s) seemed to understand me, I like the group leader(s), information was helpful, enjoyed today’s group; alphas ranged from 0.87 to 0.93); *Home practice acceptability* (5 items; same as group; alphas ranged from 0.69 to 0.93), and *Participant barriers* (subscale did not have adequate reliability and therefore was dropped; items included felt angry or irritable, felt criticized or put down by someone in group today, felt sad/down/depressed, the home practice was hard to do).

#### Acceptability overall

Participants were also asked to complete a final, summative evaluation of the acceptability of the ADAPT4U program and condition which they experienced. This questionnaire had 21 questions: seven asking general evaluation questions (e.g., “I have noticed positive changes in my children’s behavior since the beginning of program;” rated 1–5, Not at all True to Very True), 11 asking how helpful specific content areas of the program were (e.g., giving good directions; rated 1–5, Not Helpful to Very Helpful), one asking about facilitator abilities (rated 1–5, Poor to Excellent), one asking whether the participant would recommend the program to other parents (rated 1–5, Not at all to Very Much), and one asking if issues were not covered that the parent would have liked (Yes/No). We computed an overall acceptability score for each participant based on their responses to the first 18 questions (to maintain a consistent scoring scale and keep it about the program). Cronbach’s alpha = 0.79, which is considered good consistency.

### Quantitative Data Analysis

Using factor analysis and linear regression, we compared the telehealth and group formats in terms of feasibility and acceptability, as well as what participant demographics may be associated with attendance and retention in each condition. We used chi-square, t-tests, and ANOVA tests to examine the relationship between engagement (at least one session) and retention (50% of the sessions/modules), and weekly and overall acceptability with a variety of demographics variables, including sites, formats, parent sex, deployment, child age, child gender, parent age, education level, race, marital status, child relationship with parent, and family income.

Acceptability analyses were performed in IBM SPSS Statistics (Version 27), MPlus 8.8 (Muthén & Muthén (2017)), and feasibility analyses were performed using RStudio Version 2021.09.1.

### Managing Potential for Nested Data

Because there were many families with more than one parent participating, there was the potential for nested data. To address this, for feasibility/attendance data, we used any family member attending as affirmative. For acceptability, to maintain power since some families had one parent and some had both participate, we examined intra-class correlation coefficients (ICCs) and determined the need to manage nested data due to most ICCs being at 0.05 or greater. We did this using the COMPLEX feature in MPlus to adjust the standard errors.

### Qualitative Data Analysis

In addition to the quantitative survey questions in the final evaluation, participants were asked for qualitative feedback on the program and improvements they would suggest through open-ended questions on the final survey. Twenty-four participants from the group condition and 17 participants from the telehealth condition provided this data. Using inductive content analysis (Elo & Kyngäs, 2008) on qualitative survey questions, we examined the unique features of each condition that factored into participant ratings as well as suggestions for improvement of each condition. Qualitative analyses were conducted within Microsoft Excel 2016. We first determined codes, then categories, then themes related to participants’ experience and suggestions. For reliability, one author coded group data, one coded telehealth data, and a third coded both. All three coders met together to reconcile differences in codes, which were minimal, and determine wording of categories and themes.

## Results

Despite families being randomized to the different conditions, there was a difference in father’s age between the three conditions, i.e., fathers in the online condition were younger than the other two conditions. However, one-way ANCOVA analyses determined that father’s age did not affect acceptability levels.

### Completion of Acceptability Surveys

We completed simple regressions and one-way ANOVAs to assess whether acceptability data were driven by any demographic factors (see Table 2). Outcomes examined whether either parent completed any of the acceptability questionnaires (any of the weekly questionnaires or the overall acceptability questionnaire). Most of these tests were not significant; however, participants in Minnesota rather than Michigan (*F* = 4.58, *p* = 0.03), older fathers (*B* = 0.01, *p* = 0.02, *CI* = 0.002–0.026) and moms with military service were more likely to complete any acceptability questionnaire (*t* = −2.24, *p* = 0.03).

**Table 2:** Satisfaction by demographics

					95% CI	
Demographic variable	*B*	*SE*	*t*	*p*	Lower	Upper
Regression						
Parent age						
Overall satisfaction – mom	−0.01	0.01	−0.89	0.38	−0.03	0.01
Overall satisfaction – dad	0.02	0.01	1.41	0.18	−0.01	0.06
Participant completed ≥1 satisfaction survey – mom	0.01	0.01	1.05	0.29	−0.01	0.02
Participant completed ≥1 satisfaction survey – dad	0.01	0.01	2.37	**0.02**	0.00	0.03
Child age						
Overall satisfaction – mom	−0.05	0.02	−2.28	**0.03**	−0.10	−0.01
Overall satisfaction – dad	0.01	0.05	0.30	0.77	−0.08	0.12
Participant completed ≥1 satisfaction survey – combined	0.01	0.02	0.65	0.52	−0.02	0.04
Family annual income						
Overall satisfaction – mom	−0.04	0.04	−1.00	0.32	−0.12	0.04
Participant completed ≥1 satisfaction survey – mom	−0.01	0.02	−0.27	0.79	−0.05	0.04
Overall satisfaction – dad	−0.11	0.07	−1.46	0.16	−0.26	0.05
Participant completed ≥1 satisfaction survey – dad	0.04	0.03	1.52	0.13	−0.01	0.09
Times deployed overseas						
Overall satisfaction – mom	0.34	0.26	1.30	0.22	−0.23	0.90
Participant completed ≥1 satisfaction survey – mom	−0.05	0.07	−0.65	0.52	−0.20	0.10
Overall satisfaction – dad	0.12	0.07	1.67	0.12	−0.03	0.27
Participant completed ≥1 satisfaction survey – dad	−0.01	0.03	−0.26	0.80	−0.06	0.04

ANOVA			
	*df*	*F*	*p*
Site (Minnesota vs. Michigan)			
Overall satisfaction – mom	1	0.01	0.91
Overall satisfaction – dad	1	1.67	0.21
Participant completed ≥1 satisfaction survey	1	4.58	**0.03**
Child gender			
Overall satisfaction – mom	1	1.17	0.29
Overall satisfaction – dad	1	2.50	0.13
Participant completed ≥1 satisfaction survey	1	0.00	0.96
Marital status			
Overall satisfaction – mom	3	1.53	0.23
Participant completed ≥1 satisfaction survey – mom	4	1.22	0.30
Overall satisfaction – dad	2	1.56	0.24
Participant completed ≥1 satisfaction survey – dad	4	1.27	0.29
Employment			
Overall satisfaction – mom	4	1.09	0.38
Participant completed ≥1 satisfaction survey – mom	7	0.37	0.92
Overall satisfaction – dad	3	1.46	0.26
Participant completed ≥1 satisfaction survey – dad	7	0.62	0.74
Parent race			
Overall satisfaction – mom	1	2.25	0.12
Participant completed ≥1 satisfaction survey – mom	6	0.62	0.72
Overall satisfaction – dad	1	2.56	0.13
Participant completed ≥1 satisfaction survey – dad	5	1.45	0.21
Parent ethnicity			
Overall satisfaction – mom	*Insufficient data in more than one group to analyze*		
Participant completed ≥1 satisfaction survey – mom	2	0.69	0.50
Overall satisfaction – dad	1	1.80	0.20
Participant completed ≥1 satisfaction survey – dad	2	0.87	0.42
Parent education			
Overall satisfaction – mom	3	0.31	0.82
Participant completed ≥1 satisfaction survey – mom	6	1.26	0.28
Overall satisfaction – dad	3	0.57	0.65
Participant completed ≥1 satisfaction survey – dad	7	1.16	0.33
*t-test*	*df*	*t*	*p*
Military service			
Overall satisfaction – mom	33	−0.20	0.84
Participant completed ≥1 satisfaction survey – mom	217	−2.24	**0.03**
Overall satisfaction – dad	18	1.60	0.13
Participant completed ≥1 satisfaction survey – dad	189	0.43	0.67
Deployed overseas			
Overall satisfaction – mom	33	0.66	0.51
Participant completed ≥1 satisfaction survey – mom	217	1.74	0.08
Overall satisfaction – dad	18	−1.31	0.21
Participant completed ≥1 satisfaction survey – dad	189	−1.19	0.24

### Feasibility

Families differed in their attendance based on their recruitment location; families recruited from Minnesota were more likely than those recruited from Michigan to participate in at least one session (*X*^*2*^ *(1, 244)* = 7.79*, p* = 0.005) and at least 50% of sessions (*X*^*2*^ *(1, 244)* = 10.33*, p* = 0.001). Compared to families assigned to the group format, families assigned to online and telehealth conditions were more likely to attend at least one session (*X*^*2*^ = 9.31, *p* = 0.01) and at least 50% of sessions (*X*^*2*^ = 15.32, *p* < 0.001), while no significant differences were found between families in the online or telehealth formats. Across all three formats, mothers were more likely to attend a greater number of sessions (range *t* = 2.49–3.74, *p* = 0.015–<0.001). Families with a father who had been deployed overseas were less likely to attend at least one session (*X*^*2*^ = 6.54*, p* = 0.01), while families with a mother who had been deployed overseas were more likely to attend at least once (*X*^*2*^ = 6.21*, p* = 0.01) and at least 50% of sessions (*X*^*2*^ = 5.34*, p* = 0.02). However, due to the small number of non-deployed fathers (5.8% of fathers), the results should be treated cautiously. Biological mothers were more likely to attend at least 50% of sessions compared to non-biological mothers (*X*^*2*^ = 5.504, *p* = 0.02). No significant differences were found related to child gender, child age, parent age, education level, race, marital status, child biological relationship with father, and family income.

### Acceptability

Acceptability was measured for group and telehealth, by session and subscale (participant acceptability and home practice acceptability). Participants also completed an overall acceptability questionnaire at the end of the 14 weeks, in which they provided their overall perspectives on the ADAPT4U intervention and provided suggestions for improvement. After comparing the conditions, we examined associations between the scales, between acceptability and attendance, and whether there were any significant indicators of acceptability based on demographics.

#### Weekly session acceptability

Table 3 provides both descriptive and comparative results for weekly acceptability by subscale (participant and home practice acceptability). Group sessions were consistently rated as more satisfactory to participants than telehealth sessions with a small but significant difference in every week (range: *b* = −0.17–0.46, *p* = 0.07–<0.001). Weeks 5–7 reports of home practice were rated significantly more positively by group participants (range: *b* = −0.49–0.52, *p* = 0.02–0.03), with no significant differences across conditions for other weeks. Mean level weekly acceptability was significantly higher for group participants for both participant acceptability (*b* = −0.33, *p* < 0.001) and home practice acceptability (*b* = −0.29, *p* = 0.04).

**Table 3:** Participant weekly acceptability descriptive statistics and independent samples *t*-tests

	Week	Condition	*N*	*M*	*SD*	ICC	# Responses	# Families	*b*	*SE*	*p*
Participant acceptability	1	Group	55	4.44	0.46	0.08	103	81	−0.17	0.1	0.07
		Telehealth	48	4.27	0.38						
	2	Group	51	4.58	0.4	0.05	94	76	−0.19	0.08	0.02
		Telehealth	43	4.39	0.31						
	3	Group	50	4.54	0.38	0.04	92	72	−0.22	0.08	0
		Telehealth	42	4.32	0.31						
	4	Group	47	4.54	0.47	0.05	87	67	−0.2	0.09	0.02
		Telehealth	40	4.34	0.34						
	5	Group	45	4.59	0.37	0.05	81	63	−0.31	0.09	<0.001
		Telehealth	36	4.28	0.35						
	6	Group	44	4.59	0.48	0.11	82	66	−0.35	0.11	0
		Telehealth	38	4.24	0.4						
	7	Group	37	4.57	0.49	0.12	70	59	−0.37	0.12	0
		Telehealth	33	4.2	0.42						
	8	Group	44	4.69	0.34	0.09	78	61	−0.46	0.09	<0.001
		Telehealth	34	4.23	0.39						
	9	Group	33	4.63	0.38	0.08	67	58	−0.32	0.09	<0.001
		Telehealth	34	4.3	0.37						
	10	Group	34	4.64	0.41	0.2	64	54	−0.33	0.11	0
		Telehealth	30	4.3	0.34						
	11	Group	37	4.66	0.42	0.08	67	53	−0.31	0.1	0
		Telehealth	30	4.35	0.32						
	12	Group	32	4.68	0.43	0.23	60	51	−0.44	0.11	<0.001
		Telehealth	28	4.24	0.37						
	13	Group	34	4.58	0.49	0.05	62	51	−0.26	0.12	0.03
		Telehealth	28	4.32	0.4						
	Mean	Group	72	4.58	0.37	0.13	143	96	−0.33	0.06	<0.001
		Telehealth	71	4.26	0.32						
Home Practice acceptability	2	Group	45	4.91	0.64	0.09	87	73	−0.3	0.19	0.1
		Telehealth	42	4.61	0.99						
	3	Group	44	5.02	0.71	0.07	85	67	−0.06	0.18	0.73
		Telehealth	41	4.95	0.81						
	4	Group	44	5.06	0.93	0.11	84	66	−0.38	0.23	0.09
		Telehealth	40	4.68	1						
	5	Group	41	4.88	0.89	0.12	77	61	−0.49	0.23	0.03
		Telehealth	36	4.39	0.93						
	6	Group	40	5.05	0.9	0.3	78	63	−0.49	0.21	0.02
		Telehealth	38	4.57	0.92						
	7	Group	34	4.96	0.84	0.07	67	57	−0.52	0.22	0.02
		Telehealth	33	4.44	0.86						
	8	Group	40	4.69	1.13	0.28	74	59	0	0.23	0.99
		Telehealth	34	4.69	0.81						
	9	Group	28	5	0.89	0.36	60	52	−0.46	0.24	0.06
		Telehealth	32	4.55	0.93						
	10	Group	29	4.8	0.95	0.1	59	52	−0.31	0.27	0.26
		Telehealth	30	4.49	1.02						
	11	Group	35	4.79	1.09	0.33	64	51	−0.19	0.27	0.48
		Telehealth	29	4.6	1.01						
	12	Group	30	4.85	0.88	0.29	58	49	−0.15	0.23	0.51
		Telehealth	28	4.7	0.72						
	13	Group	29	4.87	0.9	0.03	56	47	0.09	0.26	0.71
		Telehealth	27	4.96	0.73						
	Mean	Group	69	4.89	0.78	0.03	135	91	−0.29	0.14	0.04
		Telehealth	66	4.6	0.69						

### Overall Acceptability with the Intervention

#### Acceptability by demographics

Table 2 provides analytic results for overall acceptability for demographics. The only significant result was that mothers who had younger children were more satisfied than mothers with older children (*B* = −0.05, *t* = −2.28, *p* = 0.03, *95% CI* = −0.10–−0.01).

#### Acceptability by condition

Table 3 provides both descriptive and comparative results for overall acceptability in the two conditions. Overall, acceptability was high in both conditions and there was little difference in acceptability levels between them. In the seven general acceptability questions, means were between 4.34–5.0 on a 5-point scale (except for one reverse-coded item). Telehealth participants rated significantly higher how helpful sessions were, that the facilitator was understanding and responsive to their situation, and that they would participate in a future parenting program based on their experience. Regarding specific aspects of the program (sessions, e.g., managing emotions, giving good directions), means were 3.77–4.86 and there were no differences between the conditions on how helpful each aspect of the program was. The final three questions asked about facilitators’ teaching abilities, recommending the program to other parents, and issues that were not discussed; again, acceptability was high (means 4.91–5.0), few parents identified issues not discussed, and there was no difference between the two conditions.

### Associations between Acceptability Scales

Correlations between the Participant Acceptability subscale and the Home Practice Acceptability subscale were *r* = 0.38, indicating a strong relationship between participants’ in-session/group acceptability and their positive feedback about the homework, including their ability to do it.

#### Qualitative survey data

Several themes emerged from the qualitative data. Table 4 shows a full list of themes and categories for both conditions, and highlights where similarities and differences lie. Overall, responses tended to fall into either the positive feedback category or recommendations to further improve the program and/or its set-up. In both group and telehealth conditions, many participants had positive experiences with their facilitators, appreciating their time and planning; unique to the telehealth condition, facilitators were able to be flexible in scheduling to accommodate family schedules and last-minute changes. For example, one participant stated, “[Facilitator] was a great facilitator and helped me along when I couldn’t get started. She was encouraging and kind.” Another parent stated, “[The online format] allowed for greater flexibility and it integrated into our lives better.” Participants also appreciated the content of the program. One father stated, “I feel like this material will be a great addition to my parenting skills and resources.” There were some suggestions for additions to the program, particularly from those in the telehealth condition; topics included co-parenting/couple communication, parenting in a digital age, and how to handle substance use. Group participants appreciated that food and childcare were provided and when the location and schedule worked for them. Much of the recommendations from this condition were around scheduling, including making the program shorter than 14 weeks, scheduling on the weekend, or having a flexible online or video option. Recommendations specific to being in a group format were to split families by kids’ age (ages could range from 4–12 years old) and offering incentives for participating in the group conversation. Telehealth participants felt positive about their at-home materials but also had recommendations for improving the materials, also around some of the guidance being for younger children, such as time outs. There was positivity around the technology chosen (Webex and a mindfulness app, *Apt.Mind*), but also several instances where internet connectivity and other technology issues made participation challenging. One parent stated, “Really enjoyed this session. Technology was a barrier. [State] has had some ridiculous storms lately and technology has been an issue all week due to storms.” Several of the telehealth participants recommended or desired childcare during their meetings with facilitators, in-person meetings, and groups with other parents, which may have been an indication that the group condition would have been a better fit for them. One parent suggested, “Maybe more groups or face to face meetings…face to face would be good to help cut down on interruptions and groups to learn from other parents.” Another stated, “… possibly decreasing the time to maybe 30 min on the phone per week. One hour seems like a big commitment especially in the evening when you have to get dinner ready, make sure homework is done and get the kids ready for the next day of school and bedtime.”

**Table 4:** Themes and categories of qualitative survey data from both group and telehealth participants

Format	Theme	Positive	Negative	Recommendations
Group	Program	Program content (7)^*^ Phone calls		Program content - clarifying question Integrate video in group More video examples^*^ Follow-up (2)^*^ Child involvement in program^*^
	Structural aspects of in-person	Food provided (2) Food (2) Location Childcare (3)	*Scheduling was hard* *Scheduling - challenging* Scheduling - too long (weeks) *Location*	*Online format* *Flexibility in delivery mode* *Location - 30* *min proximity* Food variety (3)
	Structural aspects of group			Split families by kid age Participation incentives
	Facilitators	Facilitator time Facilitator planning Facilitators (3)^*^		
	Scheduling			*Scheduling - lunch break* *Schedule - weekend (2)* Scheduling - shorten/condense (4) *Scheduling - start time* Schedule - time of year
	Dissemination/recruitment			Recruitment/dissemination communication Better recruitment communication Apps/technology
Telehealth	Program	Program content (6)^*^ Format At-home materials 1:1 format		Child involvement in program^*^ More video examples^*^ More videos online Updated videos Follow-up^*^ Format - time between sessions At-home material Ongoing access to materials Program content - co-parenting Program content - age specific Program content - digital parenting Program content- substance use Program content - couple communication
	Structural aspects of remote/1:1	Webex (4) Mindfulness app	*Internet connectivity (2)* *Technology (3)*	Mobile OS delivery *Provide childcare (2)* *In-person meetings (2)* *Group with other parents*
	Scheduling	Scheduling - facilitator flexibility Flexibility (5)	Scheduling - timing	
	Engagement	Initially skeptical-but satisfied - format (2) Initially skeptical-but satisfied - content		
	Facilitators	Facilitators (7)^*^		
	Dissemination/recruitment			Dissemination by word-of-mouth

Categories in italics were directly addressed by the alternative condition; categories with ^*^ were common across conditions. Positive, negative, and recommendation columns represent the sentiment of responses

## Discussion

Our study demonstrates that the ADAPT4U telehealth format is at least as feasible and acceptable as the original in-person group format. Parents were more likely to attend both the telehealth and online formats, which indicates a significant improvement in access. However, some of the qualitative data indicate that telehealth and online formats, while generally equal in acceptability as in-person group, may be particularly attractive to certain people or groups of people (e.g., those living far from an in-person location, those with different schedules every week) and that there is still significant value to many parents in participating in person and in group format.

Our study found overall high levels of acceptability with both in-person group and individual family telehealth delivery of the ADAPT4U program both in the weekly acceptability questions and the overall intervention. These acceptability levels did not differ significantly by demographics, although the sample overall was not representative and therefore these results cannot be generalized to all parent populations. Weekly acceptability ratings were slightly higher in the group condition, but overall acceptability was mostly equivalent, and both formats had high acceptability overall. These findings are consistent with those of Englebrektsson et al., (2023), strengthening the evidence that the in-person group format is preferred over telehealth by parents; however, our attendance and acceptability data indicate that while parents may prefer group in person, many are less likely to attend it due to significant barriers. For these families, a telehealth option would be preferred. In Leijten et al., (2024)’s meta-analysis of preventive parenting intervention studies, satisfaction trended toward online supports, which may be reflective of these groups of parents who really struggle with in-person attendance due to busy schedules, transportation issues, and other barriers. It is therefore clear from previous research and our own study that providing options for what families think will be most accessible to them is critical to high engagement.

There is some indication in the qualitative data that families may struggle with participation if they have more logistical barriers, such as no reliable transportation or an unpredictable work schedule that could prevent them from being able to attend 14 weeks of the class at the same time/day each week; for example, one in-person participant stated, “It’s a 40 min drive for me, but I was very willing to travel.” One of the telehealth participants stated, “A great thanks! to our facilitator… for accommodating our ever changing schedule; especially the literal last minute changes due to family emergencies,” which indicates if this family had been in an in-person condition, they likely would not have been able to consistently attend. This is consistent with other research showing logistical barriers and competing priorities prevent greater uptake of parenting programs (Rostad et al., 2018). Contrasting the feedback from the group and telehealth conditions, it seems likely that families with more logistical barriers may have better access and engagement with an individual family telehealth option. Yet there were some participants in both groups who indicated value in the group and in-person aspects of experience, and the quantitative data indicates a small but significant difference in (higher) acceptability for participants who were able to attend in-person group. In-person may also be the only option for some families with technology barriers, as several families in the telehealth condition reported limitations in participation at times due to internet connection and other tech issues: “Technology issues as far as internet connectivity on certain days slowed or halted a couple of sessions.” Therefore, it seems clear that the format of intervention delivery is less about one format being better than another, but rather that providing multiple formats may improve access and therefore, acceptability. Program content was not significantly different between formats and was reported as satisfactory for both conditions. Future research could consider consolidating the program content in different manners to be responsive to participant suggestions and assess whether program effectiveness is maintained.

Finally, in the telehealth condition, three separate participants indicated they were initially skeptical either of participating in a parenting program in general or of the telehealth format, but in the end were glad they did so: “At first I was disappointed to learn that we were going to be part of the online cohort. But, I found that I really enjoyed it. It allowed for greater flexibility and it integrated into our lives better.” And “While I was skeptical before starting this program, I found much of the information helpful.” Lowering the barriers to participation through meeting participants remotely may mean ambivalent parents are more willing to engage; future research could assess motivation levels prior to participation to strategically match participants to a program format that best suits their situation.

### Strengths and Limitations

The key limitation was lack of acceptability data gathered for the online program, which limits the ability to compare that condition with the telehealth and in-person conditions. However, the strength of the acceptability data lies in its variety – qualitative and quantitative, weekly and overall/final questionnaires. Another limitation is the lack of comparability between the telehealth and group-based in-person condition, which lies in the confound between group vs. individual and virtual vs in person modalities. This makes it impossible to know whether differences between these two conditions are a function of the group/individual, or virtual/face-to-face formats. Our results indicate that it may be both, as suggestions for improvement seemed to address both pieces of both formats (remote/in-person, group/individual). Strengths also include the larger sample, 244 families, while limited by the tendency for the participants to be higher-income and higher-educated, as well as mostly White. However, studies regarding satisfaction/acceptability with parenting programs across low- and middle-income countries (Branco et al., 2022) and feasibility of web-based parent training programs for low-income families (McGoron, 2018) do demonstrate high ratings as well. Additionally, while random assignment to condition is a strength of this study, several participants noted disappointment with their assignment which may have impacted initial attendance and completion rates.

## Data Availability

The data and code used in the current paper are available on request from the corresponding author.
